# Association of maternal and fetal *LEPR* common variants with maternal glycemic traits during pregnancy

**DOI:** 10.1038/s41598-017-03518-x

**Published:** 2017-06-08

**Authors:** Rong Lin, Hongfang Ju, Ziyu Yuan, Liangliang Zeng, Yuantian Sun, Zhenyu Su, Yajun Yang, Yi Wang, Li Jin

**Affiliations:** 1 Department of Biology, Hainan Medical College, Haikou, Hainan China; 2 Department of Gynaecology and Obstetrics, Taizhou People’s Hospital, Taizhou, Jiangsu China; 3 Fudan-Taizhou Institute of Health Sciences, Taizhou, Jiangsu China; 4 Ministry of Education Key Laboratory of Contemporary Anthropology and State Key Laboratory of Genetic Engineering, Collaborative Innovation Center for Genetics and Development, School of Life Sciences, Fudan University, Shanghai, China; 5 CAS-MPG Partner Institute for Computational Biology, Shanghai Institute for Biological Sciences, Chinese Academy of Sciences, Shanghai, China

## Abstract

Recent studies suggested that maternal and placental leptin receptor (LEPR) may be involved in maternal glucose metabolism in pregnancy. To identify maternal and fetal *LEPR* common variants influencing gestational glycemic traits, we performed association study of 24-28-week maternal fasting glucose, glucose 1 hour after the consumption of a 50-g oral glucose load, fasting insulin and indices of beta-cell function (HOMA-β) and insulin resistance (HOMA-IR) in 1,112 unrelated women and their children. Follow-up of 36 *LEPR* loci identified 3 maternal loci (rs10889567, rs1137101 and rs3762274) associated with fasting glucose, these 3 fetal loci associated with fasting insulin and HOMA1-IR, as well as these 3 maternal-fetal loci combinations associated with HOMA2-β. We also demonstrated association of maternal locus rs7554485 with HOMA2-β and HOMA2-IR, maternal locus rs10749754 with fasting glucose, fetal locus rs10749754 with HOMA2-IR. However, these associations were no longer statistically significant after Bonferroni correction. In conclusion, our results first revealed multiple associations between maternal and fetal *LEPR* common variants and gestational glycemic traits. These associations did not survive Bonferroni correction. These corrections are overly conservative for association studies. We therefore believe the influence of these nominally significant variants on gestational glycometabolism will be confirmed by additional studies.

## Introduction

Higher levels of maternal glucose in pregnancy are associated with adverse pregnancy outcomes including birth weight above the 90th percentile, primary cesarean delivery, neonatal hypoglycemia, and fetal hyperinsulinemia^1^. These associations occur across the full range of maternal glucose levels below those diagnostic of gestational diabetes mellitus (GDM). Gestational glycemic traits and GDM are considered to result from interaction between genetic and environmental risk factors^2, 3^. The heritability for fasting plasma glucose (FPG) levels during pregnancy is estimated to be 30–71%^3^.

The leptin receptor (LEPR), also known as obesity receptor (OB-R), is a member of the cytokine receptor family and localized centrally in hypothalamus known to be important in food intake regulation as well as in peripheral tissues, such as pancreatic beta cells^4^, muscle, adipose tissue^5^, and hepatocytes^6^. Several isoforms of membrane-bound LEPR with identical extracellular and transmembrane domains but a variable intracellular domain are expressed on the surface of a wide spectrum of cells in almost all tissues by posttranscriptional alternative RNA splicing: the long form, LEPR_L_ or OB-R_L_, with full signalling capacity is highly expressed in the hypothalamus and the multiple short, signalling-defective forms, LEPR_S_ or OB-R_S_, are ubiquitously expressed^7, 8^. Soluble LEPR (sLEPR or sOB-R) is a special isoform, circulating in complex with leptin, that lacks both transmembrane and intracellular domains^7^.

The *LEPR* gene is located on chromosome 1p31, which has been linked to an acute insulin response in Pima Indians^9^. This region is also linked to type 2 diabetes (T2DM) and post-challenge plasma glucose concentrations in an Old Order Amish population^10^. The two linkage studies suggested a link between this locus and glucose homeostasis. Mutated LEPR plays a crucial role in the pathogenesis of obesity and/or diabetes in leptin receptor-deficient db/db mice, Zucker fatty (fa/fa) rats, and Koletsky rats. There are several studies indicating associations of *LEPR* gene variations with FPG levels^11^, insulin resistance^12^, and T2DM^13^.

During pregnancy, mice heterozygous for the leptin receptor (db/+) have twofold FPG and hepatic glucose production than relative to wild-type (+/+) mothers and develops spontaneous GDM^14^, suggesting that an alteration in LEPR action may play a role in gestational glucose metabolism. Alterations of the normal *LEPR* gene may also be involved in the regulation of circulating glucose in pregnant women. However, the relationship of *LEPR* gene polymorphisms with glucose levels and related traits in pregnancy has not been investigated extensively to date.

LEPR is also localized to in the microvillous and basal membranes of placental syncytiotrophoblast^15^. LEPR_L_ is expressed exclusively in the microvillous membrane, whereas LEPR_S_ is expressed in both microvillous and basal membranes. *LEPR* expression are increased in placentas from GDM^16^. In humans, sLEPR is generated by proteolytic cleavage of LEPR_L_ and LEPR_S_^8^. In rodents, sLEPR is generated by alternative splicing specifically in large amounts in the placenta during pregnancy. The increased amounts of sLEPR bind leptin, increasing the amount of bound leptin and decreasing the clearance of leptin^17^. sLEPR have been reported to increase in the maternal circulation in human insulin-dependent diabetes mellitus (IDDM) pregnancies^18^. These discoveries have made placental LEPR a possible new factor involved in maternal glucose metabolism during pregnancy that still remains unclear.

Therefore, the aim of this study was to investigate the influence of common variants in the maternal and fetal *LEPR* gene on plasma glucose, insulin values, β cell function and insulin resistance in the fasted state as well as plasma glucose 1 hour after the consumption of a 50-g oral glucose load among pregnant women. The umbilical cord blood, placental syncytiotrophoblast and fetus share the same DNA. In this study, the DNA of umbilical cord blood was used.

## Results

**Figure 1:**
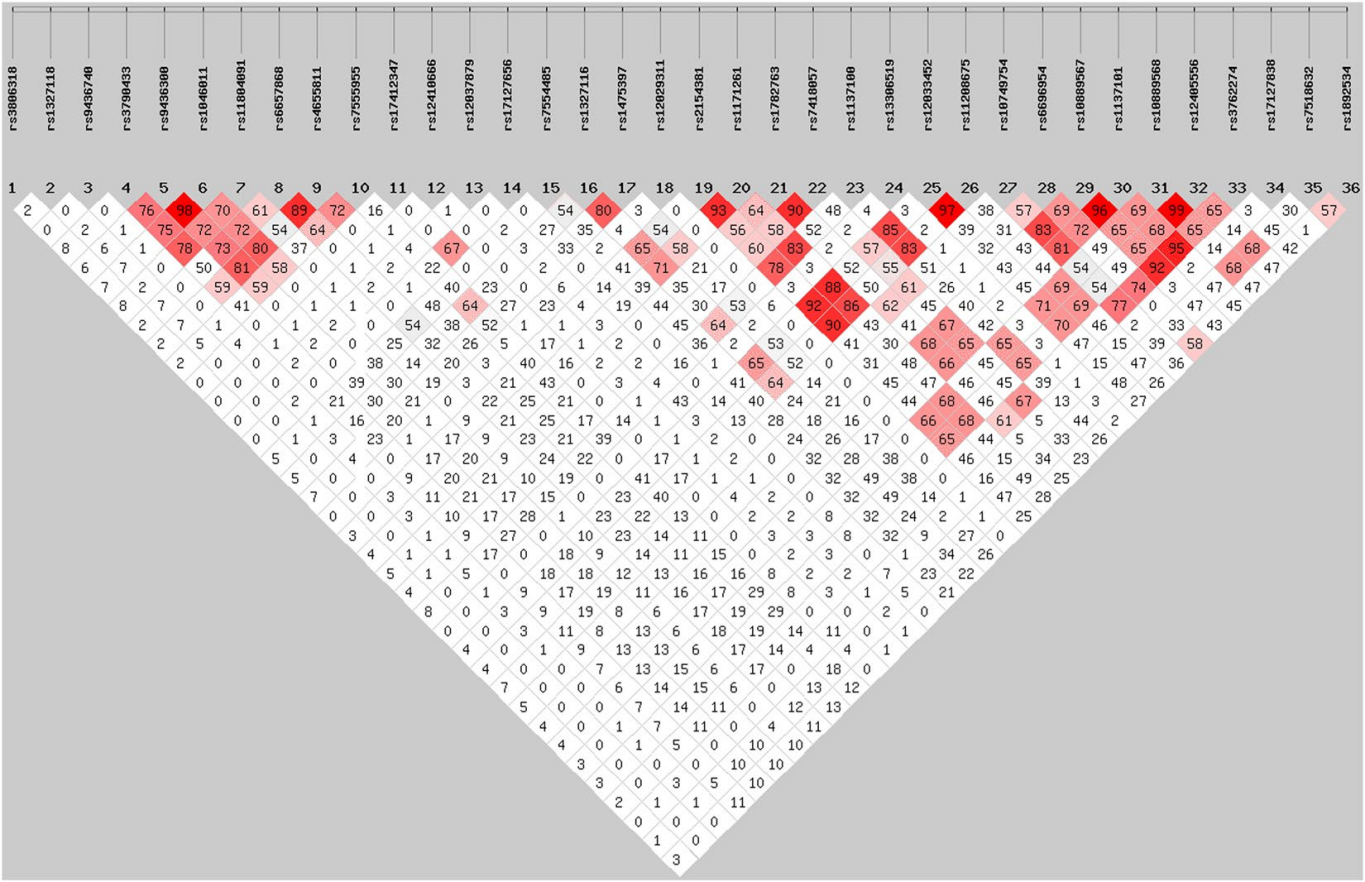
Pairwise linkage disequilibrium between SNPs, as measured by *r* ^2^ in all pregnant subjects, in the *LEPR* gene. Each diamond contains the *r* ^2^ value between the two SNPs defined by the upper left and the upper right sides of the diamond, ex: *r* ^2^ between rs3806318 (SNP 1) and rs1327118 (SNP 2) is 0.02; the redder the diamond, the higher the *r* ^2^ value. (SHEsis Software, ver. online).

The pairwise *r* ^2^ values between single nucleotide polymorphism (SNP) 5, 6 and 9 were all greater than 0.80 which indicated that SNP 5, 6 and 9 highly linked with each other (Fig. 1). The pairwise *r* ^2^ values between SNP 16 and 17, between SNP 19, 20, 23, 25 and 26, between SNP 21 and 22, between SNP 29, 30 and 33, as well as between SNP 31 and 32 were all greater than 0.80. Thus, the adjusted significant *P*-value was arbitrarily selected as 0.002 (i.e. 0.05/25) for *LEPR* since it corresponds to a corrected *P*-value of 0.05 after correction for 25 independent SNPs in the 36 SNPs analyzed in the study. All 36 SNPs that we genotyped were in Hardy-Weinberg equilibrium (HWE) with *P* > 0.05 except SNP 12, i.e. rs12410666 (*P*_maternal_ = 0.038, *P*_fetal_ = 0.018) (Supplementary Table S1). But the *P* value from the chi-square test for HWE of rs12410666 was greater than 0.002 and therefore rs12410666 was still included for further analysis. We next tested whether the genotyped SNPs were associated with gestational glycemic traits. For the sake of brevity, only the results significant at the *P* < 0.05 level are presented in Table 1. Since the SNPs in almost perfect linkage disequilibrium (LD) have almost the same effects on a phenotype, only one SNP of them is shown in Table 1.

**Table 1:** Association of *LEPR* variants with glycemic traits.

		Genotype	N	Mean(95% confidence interval)	*P*	*P* ^a^	*P* ^b^	*P* ^c^
Maternal fasting plasma glucose at 24–28 weeks’ gestation (mmol/l)								
SNP 27	rs10749754_M	0	671	4.53(4.49–4.58)	0.014	0.014	0.035^*^	0.036^*^
		1	239	4.52(4.44–4.60)				
		2	19	4.95(4.49–5.40)				
SNP 30	rs1137101_M	0	704	4.53(4.49–4.58)	0.019	0.018	0.021^*^	0.020^*^
		1	208	4.52(4.44–4.61)				
		2	17	4.95(4.44–5.46)				
SNP 2	rs1327118_F	0	517	4.54(4.49–4.59)	0.045	0.045	0.023^#^	0.023^#^
		1	164	4.64(4.52–4.75)				
		2	13	4.88(4.43–5.32)				
Maternal plasma glucose 1 hour after the consumption of a 50-g oral glucose load at 24–28 weeks’ gestation (mmol/l)								
SNP 5	rs9436300_M	0	600	7.10(6.98–7.22)	0.008	0.006	0.001^*^	0.001^*^
		1	261	7.26(7.09–7.43)				
		2	20	6.26(5.63–6.88)				
Maternal fasting plasma insulin at 24–28 weeks’ gestation (pmol/l)								
SNP 30	rs1137101_F	0	506	51.85(49.28–54.56)	0.021	0.017	0.003^#^	0.003^#^
		1	145	53.52(48.47–59.11)				
		2	5	25.43(9.88–65.47)				
SNP 33	rs3762274_MF	0	317	49.24(46.36–52.30)	0.019	0.016	0.192^*^	0.169^*^
		1	87	56.35(49.76–63.82)				
		2	57	53.15(45.42–62.19)				
		3	9	32.49(19.44–54.30)				
HOMA1-β								
SNP 14	rs17127656_M	0	782	173.65(165.25–182.47)	0.029	0.031	0.256^*^	0.210^*^
		1	76	144.73(127.40–164.41)				
SNP 35	rs7518632_M	0	514	176.41(165.82–187.68)	0.034	0.028	0.026^*^	0.028^*^
		1	304	167.44(155.08–180.77)				
		2	40	132.33(112.35–155.86)				
HOMA2-β								
SNP 15	rs7554485_M	0	700	119.45(116.46–122.52)	0.037	0.034	0.088^*^	0.094^*^
		1	139	122.69(115.85–129.93)				
		2	5	82.68(62.80–108.87)				
SNP 24	rs13306519_M	0	585	117.18(114.04–120.42)	0.009	0.007	0.454^*^	0.420^*^
		1	239	126.74(121.20–132.54)				
		2	20	113.22(95.18–134.68)				
SNP 35	rs7518632_M	0	506	121.15(117.53–124.87)	0.014	0.011	0.006^*^	0.007^*^
		1	299	119.72(115.25–124.36)				
		2	39	102.64(92.92–113.39)				
SNP 30	rs1137101_MF	0	299	112.73(108.56–117.06)	0.042	0.046	0.267^#^	0.265^#^
		1	86	121.20(113.31–129.64)				
		2	57	125.70(114.11–138.47)				
		3	8	101.50(73.34–140.48)				
HOMA1-IR								
SNP 22	rs7418057_F	0	519	1.50(1.42–1.58)	0.041	0.044	0.044^#^	0.067^#^
		1	126	1.57(1.38–1.77)				
		2	3	0.63(0.55–0.72)				
SNP 30	rs1137101_F	0	502	1.50(1.42–1.58)	0.009	0.007	0.001^#^	0.002^#^
		1	141	1.57(1.41–1.76)				
		2	5	0.66(0.25–1.71)				
SNP 33	rs3762274_MF	0	317	1.43(1.34–1.52)	0.022	0.018	0.313^*^	0.277^*^
		1	86	1.65(1.43–1.90)				
		2	55	1.56(1.33–1.85)				
		3	9	0.93(0.51–1.69)				
HOMA2-IR								
SNP 15	rs7554485_M	0	700	0.97(0.94–1.01)	0.038	0.044	0.015^*^	0.022^*^
		1	139	1.00(0.93–1.07)				
		2	5	0.58(0.35–0.96)				
SNP 22	rs7418057_F	0	499	0.97(0.93–1.01)	0.009	0.009	0.020^#^	0.026^#^
		1	123	1.00(0.91–1.10)				
		2	3	0.42(0.32–0.56)				
SNP 27	rs10749754_F	0	464	0.98(0.93–1.02)	0.025	0.028	0.059^#^	0.088^#^
		1	155	0.99(0.91–1.07)				
		2	5	0.54(0.42–0.71)				

Values presented are unadjusted arithmetic mean (95% confidence interval) for plasma glucose and geometric mean (95% confidence interval) for other variables, as well as only for variants with *P* < 0.05 in analysis of variance. For the variants in almost perfect linkage disequilibrium, only one of them is shown.

^a^Adjusted for maternal age at delivery, sex of the newborn, prepregnancy gravidity and prepregnancy parity by analysis of covariance.

^b^Adjusted for corresponding maternal or fetal variants by analysis of covariance. Adjustments for corresponding fetal variants were marked with *, and adjustments for corresponding maternal variants were marked with #.

^c^Adjusted for corresponding maternal or fetal variants, maternal age at delivery, sex of the newborn, prepregnancy gravidity and prepregnancy parity by analysis of covariance.

M: maternal genotypes, F: fetal genotypes, MF: maternal and fetal genotype combinations, 0: genotype (combination) with zero copies of the minor allele, 1: genotype (combination) with 1 copy of the minor allele, 2: genotype (combination) with 2 copies of the minor allele, 3: genotype combination with 3 copies of the minor allele, 4: genotype combination with 4 copies of the minor allele. For example, 1 for rs1137101_M indicates the maternal genotype is ‘GA’ for SNP rs1137101, 0 for rs1137101_F indicates the fetal genotype is ‘GG’ for SNP rs1137101, and 3 for rs1137101_MF indicates the genotype combination where the maternal genotype is ‘GA’ and the fetal genotype is ‘AA’, or the maternal genotype is ‘AA’ and the fetal genotype is ‘GA’, for SNP rs1137101.

### Association with maternal fasting plasma glucose at 24-28 weeks’ gestation

Four maternal *LEPR* SNPs (rs10749754, rs10889567, rs1137101 and rs3762274) were associated with 24-28-week maternal FPG: homozygous carriers of the minor allele had increased 24-28-week FPG compared with carriers of the major allele (*P* < 0.05). SNP rs10889567, rs1137101 (Gln223Arg) and rs3762274 were in high LD (all pairwise *r* ^2^ > 0.90) and thus only one of them (i.e. rs1137101) is shown in Table 1. Only one fetal *LEPR* SNP (rs1327118 in the 5′-flanking region) was associated with 24-28-week maternal FPG: subjects delivering babies homozygous for the minor allele showed highest FPG, followed by those delivering heterozygous babies, and then those delivering babies homozygous for the major allele (*P* = 0.045).

### Association with maternal plasma glucose 1 hour after the consumption of a 50-g oral glucose load at 24–28 weeks’ gestation

Significant differences were observed only among the genotype groups of two maternal *LEPR* intronic SNPs (rs9436300 and rs1046011) which were in high LD (*r* ^2^ = 0.98) (only rs9436300 shown in Table 1): homozygous carriers of the minor allele had lower plasma glucose 1 hour after the consumption of a 50-g oral glucose load as compared to carriers of the major allele (*P* < 0.01).

### Association with maternal fasting plasma insulin at 24–28 weeks’ gestation

Three fetal *LEPR* SNPs (rs10889567, rs1137101 and rs3762274) in high LD (only rs1137101 shown in Table 1) were related to maternal fasting plasma insulin: subjects delivering babies homozygous for the minor allele had almost half as much mean maternal fasting plasma insulin as those delivering babies with the major allele (*P* < 0.05). To be noted, mothers homozygous for the minor allele of the 3 SNPs had been shown to have higher 24-28-week FPG. Maternal-fetal genotype combination of SNP rs3762274 was also associated with maternal fasting plasma insulin (*P* = 0.019), but lost its significance after adjustment for fetal SNP rs3762274 (*P* = 0.192), indicating that this association was caused by the effect of fetal SNP rs3762274.

### Association with maternal homeostasis model assessment of β-cell function (HOMA-β) at 24–28 weeks’ gestation

For the minor allele frequency (MAF) of maternal *LEPR* intronic SNP rs17127656 was low (0.046), there were no minor allele homozygous mothers. Mothers with the CC genotype (major allele homozygotes) had a significantly higher HOMA1-β than those with the CT genotype (*P* = 0.029). Another two maternal *LEPR* intronic SNPs (rs7554485 and rs13306519) were associated with maternal HOMA2-β (*P* < 0.05). Mothers carrying the major allele A of *LEPR* SNP rs7518632 in the 3′-flanking region had significantly higher HOMA1-β and HOMA2-β as compared to mother homozygous for the minor allele C (*P* < 0.05). Maternal and fetal genotype combinations of 3 SNPs (rs10889567, rs1137101 and rs3762274) (only rs1137101 shown in Table 1) were associated with maternal HOMA2-β (*P* < 0.05) whereas maternal and fetal SNP rs10889567, rs1137101 and rs3762274 were not (data not shown).

### Association with maternal homeostasis model assessment of insulin resistance (HOMA-IR) at 24–28 weeks’ gestation

Mothers with the CC genotype (minor allele homozygotes) of *LEPR* intronic SNP rs7554485 had a significantly lower HOMA2-IR than those with the major allele T (*P* = 0.038). Three fetal *LEPR* SNPs (rs10889567, rs1137101 and rs3762274) (only rs1137101 shown in Table 1) were associated with maternal HOMA1-IR (*P* < 0.01): mothers of children homozygous for the minor allele had significantly lower HOMA1-IR as compared to mother of children with the major allele. Similarly, mothers of children homozygous for the minor allele of SNP rs10749754 had significantly lower HOMA2-IR as compared to mother of children with the major allele (*P* = 0.025). Fetal *LEPR* SNP rs7418057 was associated with maternal HOMA1-IR and HOMA2-IR (*P* < 0.05): mothers of children homozygous for the minor allele had significantly lower HOMA1-IR and HOMA2-IR than mother of children with the major allele. Maternal-fetal genotype combination of SNP rs3762274 was also associated with maternal HOMA1-IR (*P* = 0.022), but this association may be due to the effect of fetal SNP rs3762274, for fetal *LEPR* SNP rs3762274 was associated with maternal HOMA1-IR (*P* = 0.010).

The positive associations described above were found at the level of *P* < 0.05, and these associations remained significant after controlling maternal age at delivery, sex of the newborn, prepregnancy gravidity and prepregnancy parity. However, no significant associations were identified at *P* < 0.002. That is to say, all aforementioned associations did not pass Bonferroni correction.

When adjusted for corresponding maternal or fetal genotypes, the associations between maternal rs17127656 and HOMA1-β, between maternal rs7554485 and HOMA2-β, between maternal rs13306519 and HOMA2-β, as well as between fetal rs10749754 and HOMA2-IR lost significance. One explanation is that these effects may be not independent of corresponding maternal or fetal genotypes. But there may be other explanations. For example, the sample size for the unadjusted effect of maternal rs7554485 on maternal HOMA2-β was larger than that for the adjusted effect of maternal rs7554485 on maternal HOMA2-β. The former used all maternal samples, the latter just used mother fetus pairs; not all maternal samples had corresponding fetal samples. The decrease in the number of mother homozygous for the minor allele of rs7554485 may lead to missing effect of maternal rs7554485 on maternal HOMA2-β. Similarly, the missing effect of fetal rs10749754 on maternal HOMA2-IR may be caused by this.

## Discussion

In this study, we tested whether 36 maternal and fetal *LEPR* common SNPs were associated with gestational glycemic traits, including FPG, plasma glucose 1 hour after the consumption of a 50-g oral glucose load, fasting plasma insulin, HOMA1-β, HOMA2-β, HOMA1-IR, and HOMA2-IR in pregnant Chinese Han women and their children. At *P* < 0.05, (1) 3 SNPs (rs10889567, rs1137101 (Gln223Arg) and rs3762274) of mothers were associated with maternal FPG at 24–28 weeks’ gestation, (2) the 3 SNPs of fetuses were associated with maternal fasting plasma insulin and HOMA1-IR at 24–28 weeks’ gestation, and (3) maternal and fetal genotype combinations of the 3 SNPs were associated with maternal HOMA2-β. The 3 SNPs were in high LD (each pairwise *r* ^2^ > 0.90). Furthermore, maternal *LEPR* intronic SNP rs7554485 was associated with maternal HOMA2-β and HOMA2-IR. Maternal intronic SNP rs10749754 was associated with maternal FPG and fetal SNP rs10749754 was associated with maternal HOMA2-IR. However, these associations were no longer significant after correction for multiple hypothesis testing.

Currently, only four studies have been conducted for the association between *LEPR* polymorphisms and gestational glycemic traits or/and GDM. In the Hyperglycemia and Adverse Pregnancy Outcome (HAPO) cohort, 74 maternal variants in *LEPR* were evaluated for fasting, 1-hour, and 2-hour glucose, fasting and 1-hour C-peptide, and HbA1c levels during an oral glucose tolerance test at 24–32 weeks gestation^19^. Significant associations between maternal SNP rs1137101 (Gln223Arg) and HBA1C levels in the Thai population and 1-hour C-peptide levels in the Caucasian cohort were observed, but these associations did not remain significant after multiple testing correction. Later, a genome-wide association study (GWAS) was performed in the HAPO cohort, and again confirmed the results^20^. Another GWAS discussed the correlation between maternal gene polymorphisms and GDM and did not report any maternal *LEPR* gene polymorphisms associated with GDM at the *P* < 0.0001 level^21^. Yang *et al*.^22^ examined maternal SNP rs1137101 (Gln223Arg) and showed that it was not associated with GDM risk, plasma leptin levels, fasting insulin, HOMA1-IR and quantitative insulin sensitivity check index during 24 and 30 gestational weeks. The four studies did not investigate any fetal *LEPR* gene polymorphisms. Our study is, to our knowledge, the first observational study carried out to investigate the association between fetal *LEPR* polymorphisms and maternal-fetal genotype combinations and gestational glycemic traits.

Different polymorphisms in the *LEPR* gene have been studied in nongravid populations, albeit with unclear results. Among them, SNP rs1137101 (Gln223Arg) is studied widely. Many studies indicated that *LEPR* Gln223Arg is more or less associated with glycemic traits, although a meta-analysis of sixteen individual studies showed no association between *LEPR* Gln223Arg polymorphism and T2DM^23^. Serum leptin-binding activity has been shown to be significantly lower in carriers of the A (Gln223) allele compared with the other genotypes^24^. It is reported that *LEPR* Gln223Arg is associated with insulin sensitivity index^12^, glucose clearance^12^, fasting glucose^11, 25, 26^, insulin^27^ and leptin levels^11, 27, 28^. *LEPR* Gln223Arg conferred higher risk for altered insulin and HOMA1-IR in overweight adolescents but not in normal-weight adolescents^29^. *LEPR* Gln223Arg was associated with glucose levels in neither overweight nor normal-weight adolescents^29^. Constantin *et al*. showed that *LEPR* Gln223Arg polymorphism is associated with fasting glucose but not with fasting insulin, leptin levels, and HOMA1-IR^30^.

SNP rs10889567 is genome-wide significantly associated with sLEPR levels in European women^31^. Studies have documented inverse correlations of sLEPR levels with fasting insulin, HOMA1-IR^32^, and T2DM^33^. In our study, rs10889567, rs1137101 (Gln223Arg) and rs3762274 were in high LD. At *P* < 0.05, the 3 SNPs of mothers were related to maternal FPG at 24–28 weeks’ gestation, (2) the 3 SNPs of fetuses were related to maternal fasting plasma insulin and HOMA1-IR at 24–28 weeks’ gestation, and (3) maternal and fetal genotype combinations of the 3 SNPs were related to maternal HOMA2-β. However, none of these associations remained significant after Bonferroni correction. Of note, the A (Gln223) allele frequency of rs1137101 (Gln223Arg) is less than 0.20 in Chinese Han while greater than 0.50 in Caucasians. Glycemic traits were measured during 24–28 weeks’ gestation in our study while during 24–32 weeks’ gestation in the HAPO study. The LD structures of the *LEPR* gene between Han Chinese and Caucasian populations are obviously different (Supplementary Figure S1). Different genetic backgrounds and study designs may possibly lead to different results.

Significant association between SNP 23 (rs1137100: Lys109Arg) and FPG levels were observed in a few studies^25, 26^, but more studies has documented that SNP 23 was not associated with T2DM^13, 27, 34^, early-onset T2DM^35^, HOMA1-IR^34^, fasting glucose^34–36^, insulin^27, 34^, leptin^27^ and sLEPR levels^32^. Similarly, in our study, maternal and fetal SNP 23 as well as maternal-fetal genotype combination of SNP 23 were not associated with gestational glycemic traits (data not shown).

As was found in our study, none of the observed associations at *P* < 0.05 would have survived Bonferroni correction. One interpretation is that these associations are indeed due to chance and that the tested SNPs do not contribute to these gestational glycemic traits. However, Bonferroni correction is too conservative to apply to genetic association analysis. The nominal association observed, especially for rs10889567, rs1137101 (Gln223Arg) and rs3762274, may be real, but needs confirmation. Further testing in independent populations is needed to clarify the role of these SNPs associated with gestational glycemic traits at *P* < 0.05 in our study.

Furthermore, the sample size of our study was relatively modest. One of the reasons is the practical difficulties in sample collection from mothers and their fetuses rather than just one. The study just collected 513 mother-offspring pairs. We did not successfully gather umbilical cord blood samples for 416 mothers’ newborn children and venous blood samples for 183 newborns’ mothers. For most SNPs, the number of the genotype combination where both pregnant women and their children were homozygous for the minor allele was small, usually less than 9. The size of mother-offspring pairs may not provide sufficient statistical power to detect the associations between maternal glycemic traits and maternal-fetal gene combinations. Thus, it is required to replicate these associations in larger populations of pregnant women and their children for elucidating these results. Moreover, the interactions between maternal and fetal genes are complex, and the interactions of different genes may be different. A simple additive effect is just one of the ways, so we need more mother-offspring pairs for more detailed analysis.

In our study, tag SNPs were selected from the HapMap Project database. More variants are detected in 1000 Genomes than in HapMap. We recognized that it was a limitation of the study. More variants in 1000 Genomes are required to be evaluated.

In conclusion, we report several associations between maternal and fetal *LEPR* common SNPs and gestational glycemic traits. These associations were nominally significant before correction for multiple comparisons. These corrections are too conservative for association studies. We therefore believe the effect of these nominally significant SNPs on gestational glucose metabolism will be confirmed by further study in other populations.

## Methods

### Study population

For this study, a total of 1,112 unrelated women with a singleton pregnancy were recruited. Pregnant women who delivered at Taizhou People’s Hospital between October 2010 and June 2013 were invited to participate. All participants and their husbands were Han Chinese by self-identification. All participants were over 18 years of age. The prenatal testing for all participants was conducted mainly in this hospital. Fasting glucose and insulin blood tests, as well as 50-g 1-h glucose challenge test were performed after overnight fasting (8–14 h) during 24–28 weeks’ gestation in order to screen for GDM, and a fasting glucose level of ≥5.6 mmol/l or 50-g 1-h glucose level of ≥7.8 mmol/l was considered positive and warranted a diagnostic 75-g oral glucose tolerance test. Plasma glucose and insulin levels were determined by the hexokinase method and electrochemical luminescence immunoassay (ECLIA) method, respectively. Approximately 94.9% of participants had fasting glucose level less than 5.6 mmol/l. Subjects who received pharmacological interventions for glycaemic control before screening at 24–28 weeks’ gestation were not included.

To evaluate basal pancreatic β-cell function and insulin resistance, we used the β cell function index and fasting insulin resistance index derived from the homeostasis model assessment (HOMA) model by applying the following: HOMA1 β cell function index (HOMA1-β) = 20 × fasting insulin(μU/ml)/[fasting glucose(mmol/l) − 3.5], HOMA1 insulin resistance index (HOMA1-IR) = fasting insulin (μU/ml) × fasting glucose(mmol/l)/22.5^37^. Data on fasting insulin levels of 51 participants were missing and thus HOMA1-β and HOMA1-IR of these participants could not be calculated. HOMA1-β of another 25 participants could not be determined for they had a fasting glucose level less than 3.5 mmol/l. HOMA2-β and HOMA2-IR were calculated with HOMA2 Calculator version 2.2.3 for subjects whose plasma glucose ranged from 3.0 to 25.0 mmol/l and whose insulin ranged from 20 to 400 pmol/l^38^. HOMA2-β and HOMA2-IR data were available for 1019 of 1112 (92%) subjects. Lower HOMA-β values indicate greater β-cell dysfunction, and higher HOMA-IR values indicate greater insulin resistance (IR), as verified against gold standards (*r* = 0.5–0.7)^38–40^. Compared to the gold standard but more laborious euglycemic clamp^41^ or frequently sampled intravenous glucose tolerance test^42, 43^, HOMA is more suitable and convenient for epidemiologic studies. HOMA1-IR mostly reflects hepatic IR^44, 45^, whereas HOMA2-IR reflects both hepatic and peripheral IR^38^.

Venous blood samples were collected from mothers before or after delivery and umbilical cord blood samples were collected at delivery. Finally, a total of 1,625 blood samples (i.e. 929 samples of maternal venous blood and 696 samples of neonatal cord blood) were collected for DNA extraction, including 513 venous blood samples of mothers and 513 corresponding umbilical cord blood samples of their newborn children, 416 venous blood samples of mothers without corresponding umbilical cord blood samples of their newborn children, as well as 183 umbilical cord blood samples of newborns without corresponding venous blood samples of their mothers. The present study was approved by the Ethics Committee of Hainan Medical College and the local Ethics Committee. All individuals provided informed consent before entering the study. All methods were carried out in accordance with the approved guidelines. The clinical characteristics of all subjects are summarized in Supplementary Table S2.

### SNP selection and genotyping

A combined tag and candidate SNP method was used in selecting SNPs across the *LEPR* gene and its 5 kb up-/downstream region (Chromosome 1: 65415635–65642137 226.50 kbp, human genome reference assembly GRCh38/hg38). Only SNPs with MAF greater than 5% were included. Thirty-one tag SNPs were selected on the basis of LD patterns observed in the Han Chinese (CHB) samples genotyped as part of the International HapMap Project with a minimum *r* ^2^ of 0.9 (Supplementary Table S1). We also selected four intronic SNPs (SNP 19:rs2154381, SNP 20:rs1171261, SNP 29:rs10889567, and SNP 32:rs12405556) reported to be associated with plasma sLEPR levels at genome-wide significance level^31^, as well as one missense variant (rs1137101:Gln223Arg).

All SNPs were genotyped with a custom-by-design 48-Plex SNPscan^TM^ Kit (Cat#:G0104; Genesky Biotechnologies Inc., Shanghai, China). As depicted by Chen *et al*.^46^, the kit was based on double ligation and multiplex fluorescence polymerase chain reactions and developed according to patented SNP genotyping technology by Genesky Biotechnologies Inc. Maternal and fetal DNA samples were distributed to 96-well plates. One negative control and 5 blind duplicate samples were included in every plate. Finally, approximately 5.5% of blind samples were examined in duplicate, and the concordance rate between the samples and the blind duplicates was more than 99%. Each polymorphism was successfully assayed in ≥99.8% of the samples (Supplementary Table S1).

### Statistical analysis

HWE for the polymorphisms was assessed by a chi-squared test. The LD between all pairwise SNPs was quantitated as *r* ^2^ using the Web-based programs SHEsis (http://analysis.bio-x.cn/myAnalysis.php) and SNPStats (http://bioinfo.iconcologia.net/snpstats/start.htm). Two variants with *r* ^2^ < 0.50 was thought to be in low LD, 0.80 > *r* ^2^ > 0.50 in moderate high LD, *r* ^2^ > 0.80 in high LD and *r* ^2^ = 1 in perfect LD. Variants with pairwise *r* ^2^ < 0.80 were considered to be independent. Because several SNPs genotyped were in high LD with each other and hence were far from independent, the effective number of independent variants was considered for multiple testing correction, according to the Bonferroni method.

Before statistical analysis, fasting insulin, HOMA1-β, HOMA2-β, HOMA1-IR and HOMA2-IR were transformed to an approximately normal shape by taking the natural logarithm of each value. Analysis of variance (ANOVA) was used for comparison of maternal glycemic traits among genotype groups. If the mother fetus pair had exactly three or four copies of a specific allele, this combination may increase risk for high or low maternal glycemic traits. Therefore we also compared maternal glycemic traits among maternal-fetal genotype combination groups, in addition to maternal and fetal genotype groups, for each SNP. Analysis of covariance (ANCOVA) was performed to adjust for maternal age at delivery, sex of the newborn, prepregnancy gravidity and prepregnancy parity.

In order to test whether the effect of maternal genotypes on maternal glycemic traits was influenced by the effect of corresponding fetal genotypes, the effect of maternal genotypes on maternal glycemic traits was also adjusted for corresponding fetal genotypes as classification variables in ANCOVA. Similarly, the effect of fetal genotypes on maternal glycemic traits was also adjusted for corresponding maternal genotypes as classification variables in ANCOVA. For example, the effect of fetal rs1137101 genotypes on maternal HOMA1-IR was adjusted for maternal rs1137101 genotypes. All *P* values were derived from two-sided statistical tests. These statistical analyses were implemented in Statistical Package for Social Science (SPSS version 15.0; Chicago, IL, USA).

## Electronic supplementary material

Supplementary Information
