# Supplementary material for: Association of maternal and fetal LEPR common variants with maternal glycemic traits during pregnancy
Source: Sci Rep. 2017 Jun 8;7:3112. doi: 10.1038/s41598-017-03518-x (PMC5465219; doi:10.1038/s41598-017-03518-x)
Supplement: Supplementary file 1 — Supplementary Information [file 41598_2017_3518_MOESM1_ESM.pdf]

Association of maternal and fetal *LEPR* common variants with maternal glycemic traits during pregnancy

Rong Lin[1,*], Hongfang Ju[2], Ziyu Yuan[3,4], Liangliang Zeng[1], Yuantian Sun[1], Zhenyu Su[1], Yajun Yang[3,4], Yi Wang[4] & Li Jin[3,4,5]

No.	rs ID	Position (bp)	Minor/Major allele	Region	Call rate	HWE (*P* value)(maternal)	HWE (*P* value)(fetal)	MAF (maternal)	MAF (fetal)
1	rs3806318	Chr 1: 65419674	G/A	5'-flanking	0.999	0.264	0.376	0.121	0.123
2	rs1327118	Chr 1: 65419886	G/C	5'-flanking	0.998	0.839	0.999	0.134	0.137
3	rs9436740	Chr 1: 65426218	T/A	intron	1.000	0.931	0.683	0.165	0.176
4	rs3790433	Chr 1: 65428659	T/C	intron	0.999	0.264	0.986	0.156	0.156
5	rs9436300	Chr 1: 65429581	G/A	intron	1.000	0.161	0.982	0.171	0.185
6	rs1046011	Chr 1: 65433313	C/T	intron	0.999	0.228	0.828	0.171	0.186
7	rs11804091	Chr 1: 65438196	G/A	intron	1.000	0.868	0.143	0.133	0.140
8	rs6657868	Chr 1: 65448024	G/A	intron	0.999	0.387	0.672	0.136	0.149
9	rs4655811	Chr 1: 65457474	G/C	intron	0.998	0.237	0.414	0.149	0.165
10	rs7555955	Chr 1: 65471796	G/A	intron	1.000	0.691	0.594	0.173	0.189
11	rs17412347	Chr 1: 65472578	T/C	intron	0.999	0.292	0.336	0.033	0.035

12	rs12410666	Chr 1: 65472986	T/C	intron	1.000	0.038	0.018	0.077	0.080
13	rs12037879	Chr 1: 65477024	A/G	intron	0.999	0.192	0.051	0.182	0.190
14	rs17127656	Chr 1: 65477788	T/C	intron	0.999	0.144	0.524	0.046	0.051
15	rs7554485	Chr 1: 65480223	C/T	intron	0.999	0.546	0.744	0.090	0.091
16	rs1327116	Chr 1: 65510368	C/A	intron	1.000	0.656	0.582	0.125	0.128
17	rs1475397	Chr 1: 65517475	T/C	intron	0.999	0.964	0.529	0.104	0.104
18	rs12029311	Chr 1: 65517667	A/G	intron	1.000	0.784	0.808	0.249	0.256
19	rs2154381*	Chr 1: 65530018	A/G	intron	0.999	0.958	0.787	0.157	0.156
20	rs1171261*	Chr 1: 65535719	C/T	intron	0.999	0.805	0.735	0.164	0.162
21	rs1782763	Chr 1: 65542217	T/C	intron	0.999	0.776	0.330	0.113	0.111
22	rs7418057	Chr 1: 65558024	A/G	intron	1.000	0.822	0.159	0.107	0.103
23	rs1137100	Chr 1: 65570758	A/G	Lys109Arg	1.000	0.351	0.375	0.180	0.180
24	rs13306519	Chr 1: 65572246	G/C	intron	1.000	0.500	0.908	0.167	0.180

25	rs12033452	Chr 1: 65572752	C/A	intron	0.999	0.949	0.595	0.158	0.160
26	rs11208675	Chr 1: 65579741	T/G	intron	0.999	0.406	0.917	0.159	0.163
27	rs10749754	Chr 1: 65588957	G/A	intron	0.999	0.670	0.038	0.149	0.133
28	rs6696954	Chr 1: 65589312	T/G	intron	0.998	0.238	0.134	0.098	0.086
29	rs10889567*	Chr 1: 65591367	T/C	intron	0.999	0.739	0.129	0.135	0.122
30	rs1137101*	Chr 1: 65592830	A/G	Gln223Arg	1.000	0.720	0.160	0.130	0.119
31	rs10889568	Chr 1: 65593762	T/C	intron	0.999	0.908	0.465	0.178	0.168
32	rs12405556*	Chr 1: 65597434	G/T	intron	1.000	0.765	0.470	0.179	0.168
33	rs3762274	Chr 1: 65598430	T/C	intron	0.998	0.503	0.191	0.126	0.117
34	rs17127838	Chr 1: 65635592	T/G	3'-flanking	1.000	0.333	0.179	0.082	0.082
35	rs7518632	Chr 1: 65637667	C/A	3'-flanking	0.999	0.969	0.634	0.224	0.212
36	rs1892534	Chr 1: 65640261	C/T	3'-flanking	1.000	0.405	0.610	0.143	0.129

Supplementary Table S1. Characteristics of the 36 variants in *LEPR*. *Candidate SNPs. #Positions based on the human genome assembly

Build 38.

Characteristic	N	
Maternal age at delivery (years)	1109	26.7±3.8
Paternal age at delivery (years)	1106	28.1±4.4
Sex of the newborn (males/females, %)	1110	51.9%/48.1%
Prepregnancy Gravidity	1110	0.7±1.0
Prepregnancy Parity	1110	0.2±0.4
Prepregnancy BMI (kg/m^2)	1092	20.7±2.6
Maternal fasting plasma glucose at 24-28 weeks' gestation (mmol/l)	1112	4.53±0.61
Maternal plasma glucose (1 hour after the consumption of a 50-g oral glucose load) at 24-28 weeks' gestation (mmol/l)	1059	7.11±1.44
Maternal fasting plasma insulin at 24-28 weeks' gestation (pmol/l)	1061	65.19±75.94
HOMA1-β	1036	233.83±263.85
HOMA1-IR	1036	1.94±2.62
HOMA2-β	1019	128.31±48.13
HOMA2-IR	1019	1.10±0.61

Supplementary Table S2. Baseline characteristics of study participants. Data are arithmetic mean±standard deviation or percentages. BMI, body mass index; HOMA-β, homeostasis model assessment of β-cell function; HOMA-IR, homeostasis model assessment of insulin resistance.

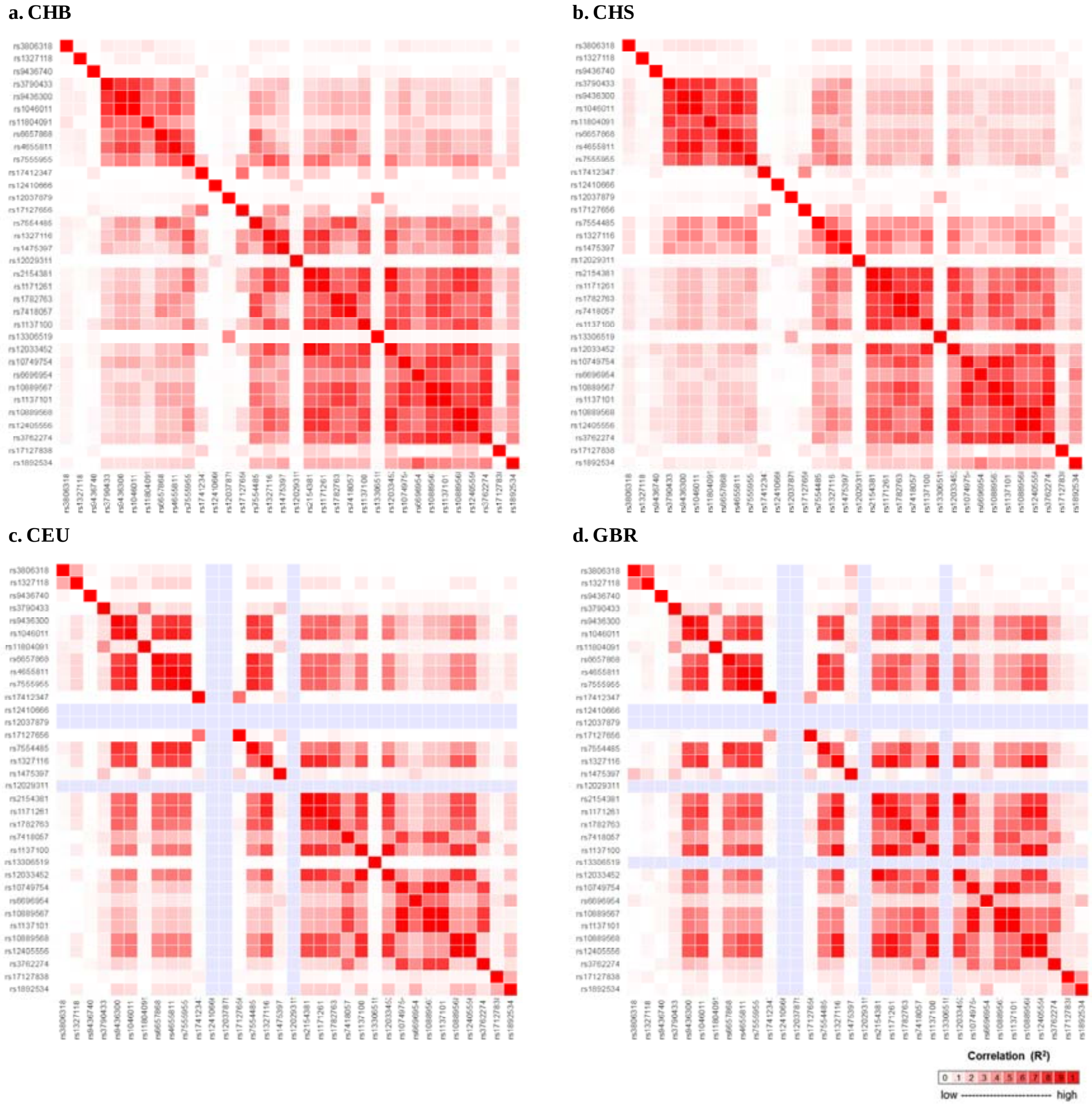

Supplementary Figure S1. Linkage disequilibrium (LD) plot of the 36 *LEPR* SNPs in Han Chinese in Bejing (CHB) (a), Southern Han Chinese (CHS) (b), Utah Residents with Northern and Western Ancestry (CEU) (c) and British in England and Scotland (GBR) (d). The LD between SNP pairs was calculated as r^2 using the Web tool LDlink 2.0 (https://analysistools.nci.nih.gov/LDlink/?tab=home) based on the phase 3 data set of the 1000 Genomes Project. The redder square represented the higher LD. It indicates that (1) the LD structures of the *LEPR* gene in two Han Chinese populations (CHB and CHS) are similar, (2) the LD structures of the *LEPR*

gene in two typical representative populations of Caucasians (CEU and GBR) are also similar, while (3) the LD structures of the *LEPR* gene between Han Chinese and Caucasian populations are obviously different.
